# A Loss to the West Ham Hospital

**Published:** 1911-09-30

**Authors:** 


### A Loss to the West Ham Hospital.

The death is announced of Dr. Walter Atkins Grogono, of Stratford, in his sixty-third year. He was a prominent surgeon in Stratford and West Ham, and was on the surgical staff of the West Ham Hospital. Besides this he held many public appointments and was Police Surgeon to the K Division. Dr. Grogono has been associated nearly all his life with the populous eastern districts of London, and as a student he began medical work at the London Hospital, qualifying in 1872 at the Royal College of Surgeons and the Apothecaries' Hall, but afterwards taking a diploma in Edinburgh in 1878. He was a Fellow of the Hunterian Society.

